# Comparison of machine and deep learning for the classification of cervical cancer based on cervicography images

**DOI:** 10.1038/s41598-021-95748-3

**Published:** 2021-08-09

**Authors:** Ye Rang Park, Young Jae Kim, Woong Ju, Kyehyun Nam, Soonyung Kim, Kwang Gi Kim

**Affiliations:** 1 Department of Health Sciences and Technology, Gachon Advanced Institute for Health Sciences and Technology (GAIHST), Gachon University, Incheon, Republic of Korea; 2 Department of Biomedical Engineering, Gachon University College of Medicine, Gil Medical Center, Incheon, Republic of Korea; 3 Department of Obstetrics and Gynecology, Ewha Womans University Seoul Hospital, Seoul, Republic of Korea; 4 Department of Obstetrics and Gynecology, Bucheon Hospital, Soonchunhyang University, Bucheon, Republic of Korea; 5 R&D Center, NTL Medical Institute, Yongin, Republic of Korea

**Keywords:** Computer science, Cancer screening, Cancer imaging, Reproductive signs and symptoms, Imaging techniques

## Abstract

Cervical cancer is the second most common cancer in women worldwide with a mortality rate of 60%. Cervical cancer begins with no overt signs and has a long latent period, making early detection through regular checkups vitally immportant. In this study, we compare the performance of two different models, machine learning and deep learning, for the purpose of identifying signs of cervical cancer using cervicography images. Using the deep learning model ResNet-50 and the machine learning models XGB, SVM, and RF, we classified 4119 Cervicography images as positive or negative for cervical cancer using square images in which the vaginal wall regions were removed. The machine learning models extracted 10 major features from a total of 300 features. All tests were validated by fivefold cross-validation and receiver operating characteristics (ROC) analysis yielded the following AUCs: ResNet-50 0.97(CI 95% 0.949–0.976), XGB 0.82(CI 95% 0.797–0.851), SVM 0.84(CI 95% 0.801–0.854), RF 0.79(CI 95% 0.804–0.856). The ResNet-50 model showed a 0.15 point improvement (*p* < 0.05) over the average (0.82) of the three machine learning methods. Our data suggest that the ResNet-50 deep learning algorithm could offer greater performance than current machine learning models for the purpose of identifying cervical cancer using cervicography images.

## Introduction

Cervical cancer is the second most common cancer in women worldwide and has a mortality rate of 60%. Currently, about 85% of the women who lose their lives to cervical cancer each year live in developing countries, where medical care is far more limited both in terms of available professionals and access to technology^1–3^. Many of these deaths could be prevented with access to regular screening tests, which would enable the effective treatment of precancer stage lesions^4,5^. As cervical cancer has no overt signs during the early stages of progression and a long latent period, early detection through regular checkups is vitally important.^6,7^.

The typical method of identifying cervical cancer is^8^ cervicography, a process in which morphological abnormalities of the cervix are determined by human experts based on cervical images taken at maximum magnification after applying 5% acetic acid to the cervix^9^. However, this method is limited in that it requires sufficient human and material resources; accurate reading of cervical dilatation tests requires a licensed professional reader and professional photography equipment capable of magnifying more than 50 times is needed in order to perform the procedure^10^. In addition, reader objectivity is limited and can only be increased through systematic and regular reader quality controls. As it stands, there likely exists inter-intra observer errors, though without systemic controls in place, data on this topic is scarce. Not only that, results can also vary depending on the subjective views of the readers and reader’s reading environment^11,12^.

To compensate for these shortcomings, computer-aided diagnostic tools such as classic machine learning (ML) and deep learning (DL) have been used to recognize patterns useful for medical diagnosis^13,14^. ML is considered a high-level construct of DL, and it refers to a series of processes that analyze and learn data before making decisions based on the learned information^15^. ML requires a feature engineering process that eliminates unnecessary variables and pre-selects only those that will be used for learning. This process is disadvantaged by the requirement that experienced professionals pre-select critical variables. Conversely, DL overcomes this shortfall by a process in which the system learns important features without pre-selected variables and the human assumptions pre-selected variables inherently includes^16^. In this paper, ML and DL concepts are used separately.

In the 2000s, ML-based cervical lesion screening techniques began to be actively studied^17^. In 2009, an artificial intelligence (AI) research team in Mexico conducted a study to classify negative images, which are those clearly without indications of cancer, and positive images, which are those judged to require close examination using the k-nearest neighbor algorithm (K-NN). K-NN has had moderate success in past studies; using images from 50 patients, k-NN was able classify negative and positive images with a sensitivity of 71% and a specificity of 59%^18^.

In another study of ML classification performed by an Indonesian group in 2020, image processing was applied to cervicography images and a classification of normal negative and abnormal positive images was conducted using a support vector machine (SVM) with an accuracy of 90%^19^.

In the field of cervical research, many research teams around the world have begun to focus on DL methods for the detection and classification of cancer^20^. In 2019, a group at Utah State University in the United States used a faster region convolution neural network (F-RCNN) to automatically detect the cervical region in cervicography images and classify dysplasia and cancer with an AUC of 0.91^21^. In 2017, a research group in Japan conducted an experiment in which 500 images of cervical cancer were classified into three grades [severe dysplasia, carcinoma in situ (CIS), and invasive factor (IC)] using the research team's self-developed neural network which showed an accuracy of about 50% during the early stages of development^22^. In 2016, Kangkana et al. conducted a study classifying Pap smear images using various models including deep convolution neural networks, convolution neural networks, least square support vector machines, and softmax regression with up to 94% accuracy^23^. ML and DL models are still being actively studied for the classification of medical images, especially for the purpose cervical lesion screenings.

In this study, we classified cervicography images as negative or positive for cervical cancer using ML and DL techniques in the same environment in order to compare their performance. ML, which requires variables pre-selected by humans to perform classification tasks, is a method that uses previously known diagnostic criteria as variables, such as morphology or texture. Conversely, DL extracts what the system identifies as critical variables through the training algorithm itself without the assumptions inherent in previous human analyses. Herein, we compare to performance of ML using previously determined diagnostic variables to that of DL, which extracts new statistical information potentially unknown to human experts. This comparison will allow future researchers to better choose which models are most suitable for their purposes, which will ultimately provide clinicians systems capable of accurately assisting in the diagnosis of cervical cancer.

## Methods

### Development environments

The neural network models were developed on the Ubuntu 18.04 OS and four NVIDIA GeForce RTX 2080 TI’s were used to train the models. With respect to graphic drivers, a 440.33 version of linux (64-bit) released in November 2019, a CUDA 10.2 version, and a CUDNN 7.6.5 version were used. Python 3.7.7 was the programming language used with the Keras 2.3.1 library based on tensorflow 1.15.0. For feature extraction, pyradiomics 3.0 developed at Harvard Medical University and the scikit-learn 0.23.1 module developed by Fabian Pedregosa's research team were used.

### Data description

The Institutional Review Board of Ewha Womans University Mokdong hospital approved (IRB No. EUMC 2015-10-004) this retrospective study and waived the requirement for informed consent for both study populations. All methods were performed in accordance with the relevant guidelines and regulations in compliance with the Declaration of Helsinki. A total of 4119 cervicography images were taken with one of three Cervicam instruments. The sizes of images obtained from each equipment differed: 1280 × 960 pixels with the Dr.Cervicam, 1504 × 1000 pixels with the Dr.Cervicam WiFi, and 2048 × 1536 pixels with the Dr.Cervicam C20. All equipment provided a cervical magnification examination video that magnified the cervix by about 50x. The grades given by the cervical magnification test are generally classified into negative and positive. The negative group includes those with no visible lesions or symptoms of cervical cancer, while the positive group refers to those clearly containing lesions in which cancerous tissue is either visible to the naked eye or that the human expert suspects has the possibility to develop into cancer. The dataset contained 1,984 normal negative images and 2,135 abnormal positive images and all data were verified as accurate via tissue biopsy. A total of 2636 images (1270 negative and 1366 positive) were used as the training set and a total of 659 images (317 negative and 342 positive) were used for validation. The test set consisted of a total of 824 images (397 negative and 427 positive).

### Data pre-processing

Cervicography images were generally wide compared to their heights. The cervical area was located in the center of the image and the vaginal wall was often photographed on the left and right sides. In the ML feature analysis stage, the entire input area is screened for feature extraction. Thus, it is typical to remove the areas outside the target area to prevent the accumulation and extraction of unnecessary data. In our dataset, providing that the cervical region was appropriately centered, the left and right ends were cropped such to make the image size uniform and to set the width equal to height. Likewise, for the DL model, the same pre-processed images were used as the input in order to create comparable conditions.

### Study design for ML analysis

**Figure 1:**
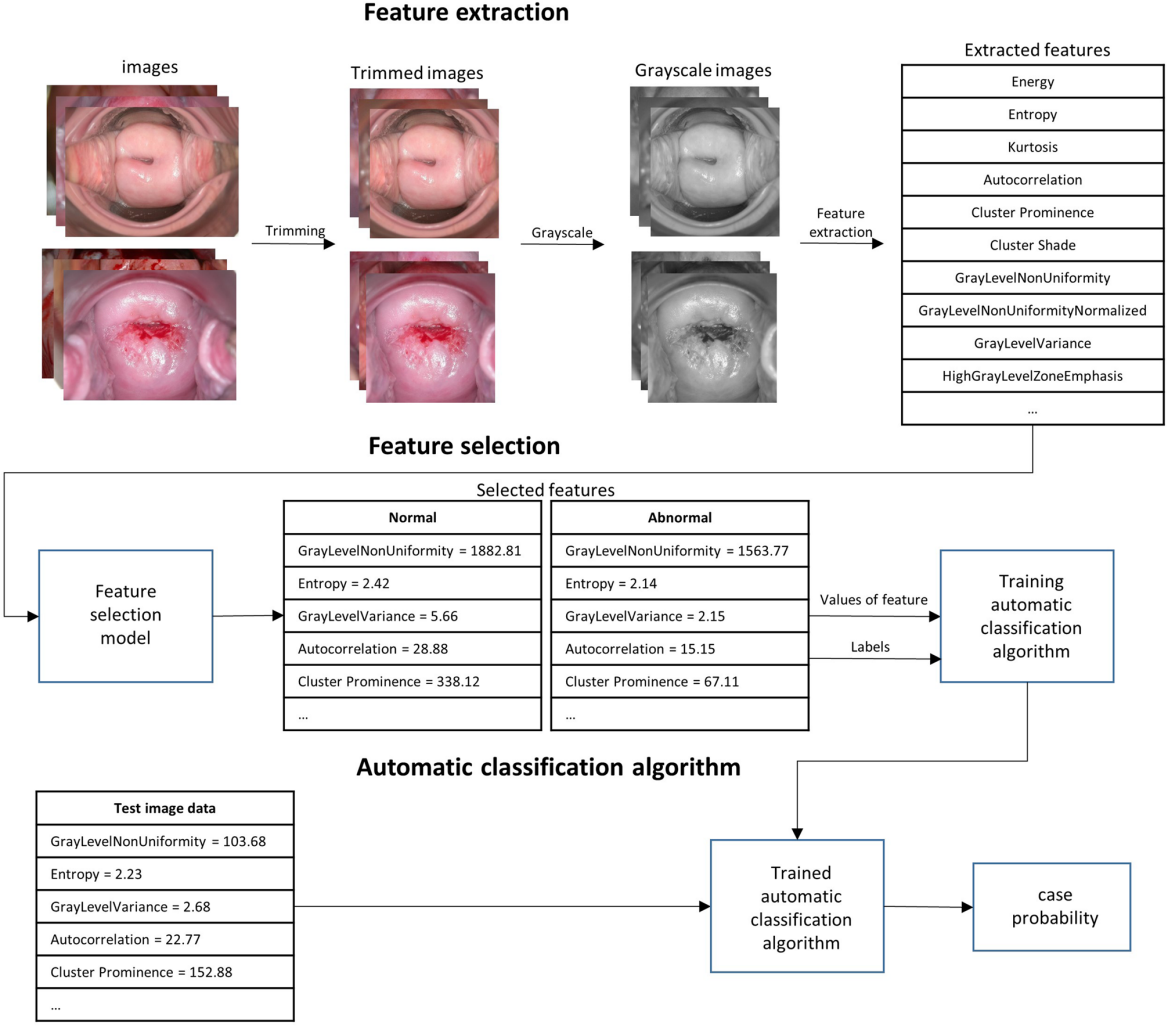
ML model training process for cervical cancer classification. The trimmed and grayscaled input data, negative and positive groups, are used to extract radiomics features. After the feature selection process, 10 features were selected for cervical cancer classification. The trained model then classified the test images and produced results.

The overall process of ML is shown in Fig. 1. Training sets were pre-processed images as described above. After extracting more than 300 features from the pre-processed images in the feature extraction stage, only the major variables affecting classification were selected via the Lasso model. In ML models, we used the Extreme Gradient Boost (XGB), Support Vector Machine (SVM), and Random Forest (RF) to train classifications from the selected variables. After training the models, a fivefold cross-validation was performed using the test set to evaluate model performance.

Eighteen first order features were identified and 24 Grey Level Co-occurrence Matrix (GLCM), 16 Grey Level Run Length Matrix (GLRLM), 16 Grey Level Size Zone Matrix (GLSZM), and 226 Laplacian of Gaussian (LoG)-filtered-first-order features were included as second order features. A total of 300 features from five categories were extracted from the training set images^24^.

A first-order feature is a value that relies only on each individual pixel value for analyzing one-dimensional characteristics such as mean, maximum, and minimum. Building upon first-order features, the GLCM of second-order-features is a matrix that takes into account the spatial relationship between the reference pixel and the adjacent pixels. Adjacent pixels refer to pixels located either east, northeast, north, and northwest from the reference pixel. Then, the second-order feature GLRLM matrix calculates how continuous pixels have the same value within a given direction and length. GLSZM identifies nine adjacent pixel zones, creating a matrix that calculates how continuous pixels with the same value are. Finally, LoG-filtered-first-order is a method of applying the Laplacian of Gaussian (LoG) filter and selecting first order features. The LoG filter is the application of a Laplaceian filter after smoothing the image with a Gaussian filter, a technique commonly used to find contours, which can be thought of as points around which there are rapid changes in the image.

ML generally adopts only key features among extracted features so that creates easy-to-understand, better-performing, and fast-learning models. The lasso feature selection method using L1 regularization is commonly used to create training data for these models, where only a few important variables are selected and the coefficients of all other variables are reduced to zero. This method is known to be simpler and more accurate than other methods of feature selection and thus is often used to select variables^25^.

The 10 features selected by lasso feature selection are as follows: Variance, Run length nonuniformity (RLN), Long run high gray level emphasis (LRHGLE), Long run emphasis (LRE), Gray level nonuniformity (GLN), Large area emphasis (LAE), Large area low gray level emphasis (LALGLE), Zone variance (ZV), Size zone nonuniformity (SZN) and LoG-sigma-10 mm 3D Energy (Table 1).

**Table 1:** Definition of the 10 ML features.

Features	Definition	Description
**Histogram**		
Variance	$\frac{1}{{N}_{p}}\sum_{i=0}^{{N}_{p}}{(X\left(i\right)-\overline{X })}^{2}$	The mean of squared distances of each intensity value
**GLRLM**		
Run length nonuniformity (RLN)	$\frac{{\sum }_{j=1}^{{N}_{r}}{({\sum }_{i=1}^{{N}_{r}}P(i,j|\theta ))}^{2}}{{N}_{r}(\theta )}$	The nonuniformity of the run length
Long run high gray level emphasis (LRHGLE)	$\frac{{\sum }_{i=1}^{{N}_{g}}{\sum }_{j=1}^{{N}_{r}}P(i,j|\theta ){i}^{2}{j}^{2}}{{N}_{r}(\theta )}$	The joint distribution of long run length
Long run emphasis (LRE)	$\frac{{\sum }_{i=1}^{{N}_{g}}{\sum }_{j=1}^{{N}_{r}}P(i,j|\theta ){j}^{2}}{{N}_{r}(\theta )}$	The distribution of the long run length
**GLSZM**		
Gray level nonuniformity (GLN)	$\frac{{\sum }_{i=1}^{{N}_{g}}{({\sum }_{j=1}^{{N}_{s}}P(i,j))}^{2}}{{N}_{z}}$	The variability of gray-level intensity values in the image
Large area emphasis (LAE)	$\frac{{\sum }_{i=1}^{{N}_{g}}{\sum }_{j=1}^{{N}_{s}}P(i,j){j}^{2}}{{N}_{z}}$	The distribution of large area size zones
Large area low gray level emphasis (LALGLE)	$\frac{{\sum }_{i=1}^{{N}_{g}}{\sum }_{j=1}^{{N}_{s}}\frac{P(i,j){j}^{2}}{{i}^{2}}}{{N}_{z}}$	The proportion in the image of the joint distribution of larger size zones
Zone variance (ZV)	${\sum }_{i=1}^{{N}_{g}}{\sum }_{j=1}^{{N}_{s}}P(i,j){(j-\mu )}^{2}$	The variance in zone size volumes for zones
Size zone nonuniformity (SZN)	$\frac{{\sum }_{j=1}^{{N}_{s}}{({\sum }_{i=1}^{{N}_{g}}P(i,j))}^{2}}{{N}_{z}}$	The variability of size zone volume in the image
**LoG filtered first order**		
LoG sigma 10 mm 3D Energy	${V}_{voxel}\sum_{i=0}^{{N}_{p}}{(X\left(i\right)+c)}^{2})$	Energy values in three dimensions with LoG filters

### ML classification architectures

**Figure 2:**
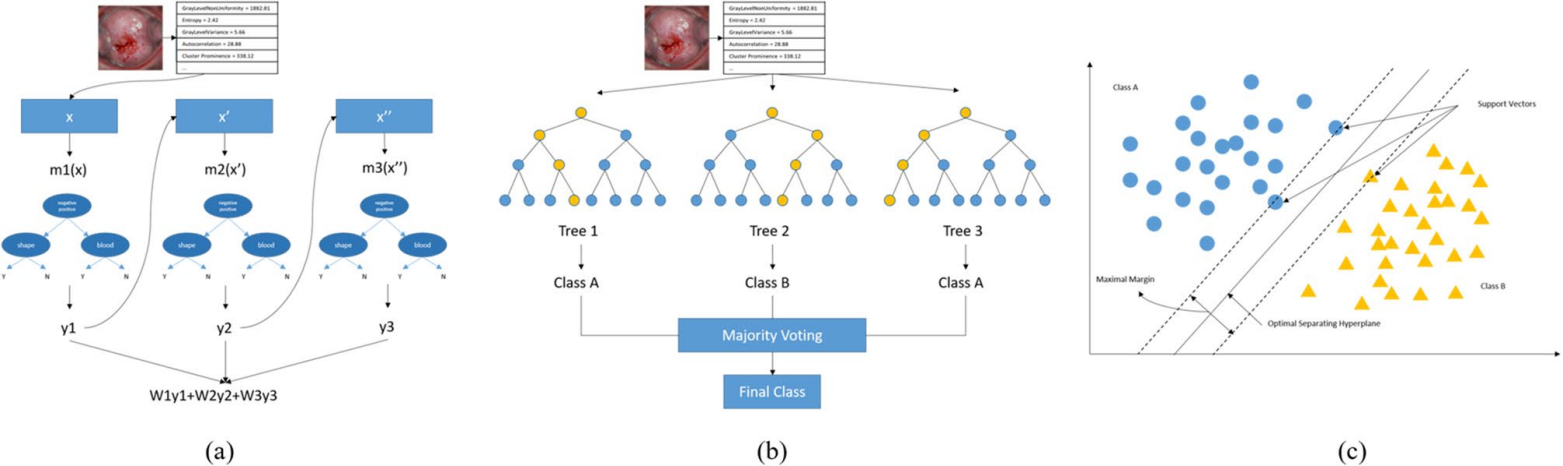
The diagrams of ML model architectures for cervical cancer classification. (**a**) In XGB, the y value extracted from one feature is used as an input value to predict the next feature, and the final y values after this process are combined with the weights. (**b**) RF creates several decision trees and synthesizes the results of each tree to determine the final class. (**c**) After linearly classifying the data, SVM finds the boundary with the largest width through an algorithm.

For ML classification, we used the XGB, RF, and SVM architectures. XGB is a boosting method that combines weak predictive models to create strong predictive models^26^. As shown in Fig. 2a, a pre-pruning method is used to compensate for the error of the previous tree and create the next tree. The RF model as shown in Fig. 2b is a method of bagging. After a random selection of variables, multiple decision trees are created. The results are then integrated into an ensemble technique that is used to classify data^27^. SVM is a linear regression method; when classifying two classes as shown in Fig. 2c, it finds data closest to the line called the vector and then selects the point where the margin of the line and support vector maximizes^28^. With all of these models, a default value is designated for each parameter. Default values for the main parameters of the XGB model are: loss function = linear stress; evaluation metric = RMSE; learning rate eta = 0.3; and max default = 6. The SVM's main parameter default values are: normalization parameter C = 1, the kernel used by the algorithm = RBF, and the kernel factor gamma = 1. Default values for the key parameters of the RF model are: number of crystal n-estimators = 100, the classification performance evaluation indicator = ‘gini’, max depth of the crystal tree = None, and the minimum number of samples required for internal node division = 2.

### Study design for DL analysis

**Figure 3:**
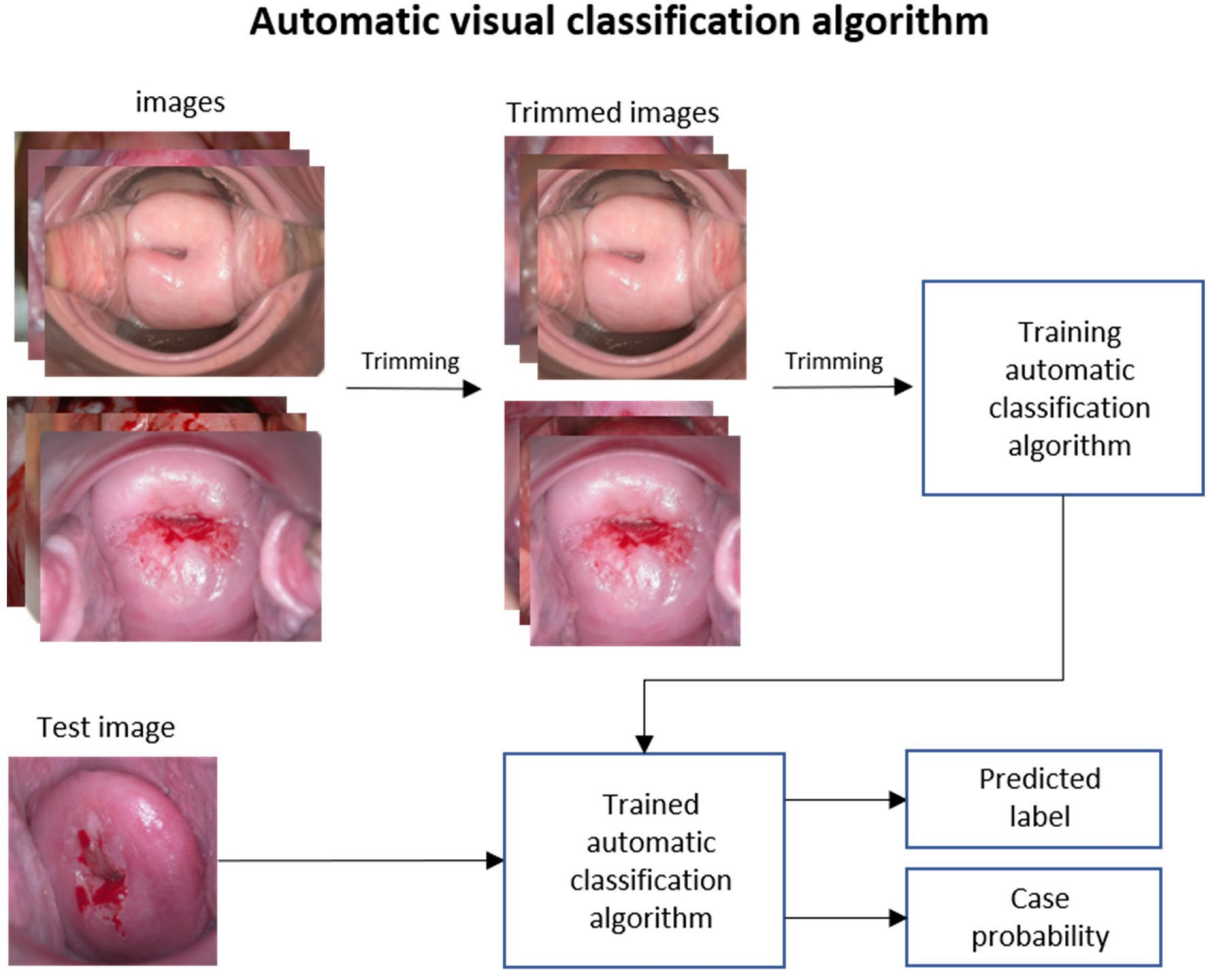
DL model training process for cervical cancer classification. The input images are trimmed and used as training data. The trained model then predicts test data.

The entire DL process is shown in Fig. 3. After preprocessing images as was done for the ML models, the model was created based on the ResNet-50 architecture. The generated model was then applied to the test set and the performance of the ML and DL models was evaluated through fivefold cross-validation.

### DL classification architecture

**Figure 4:**
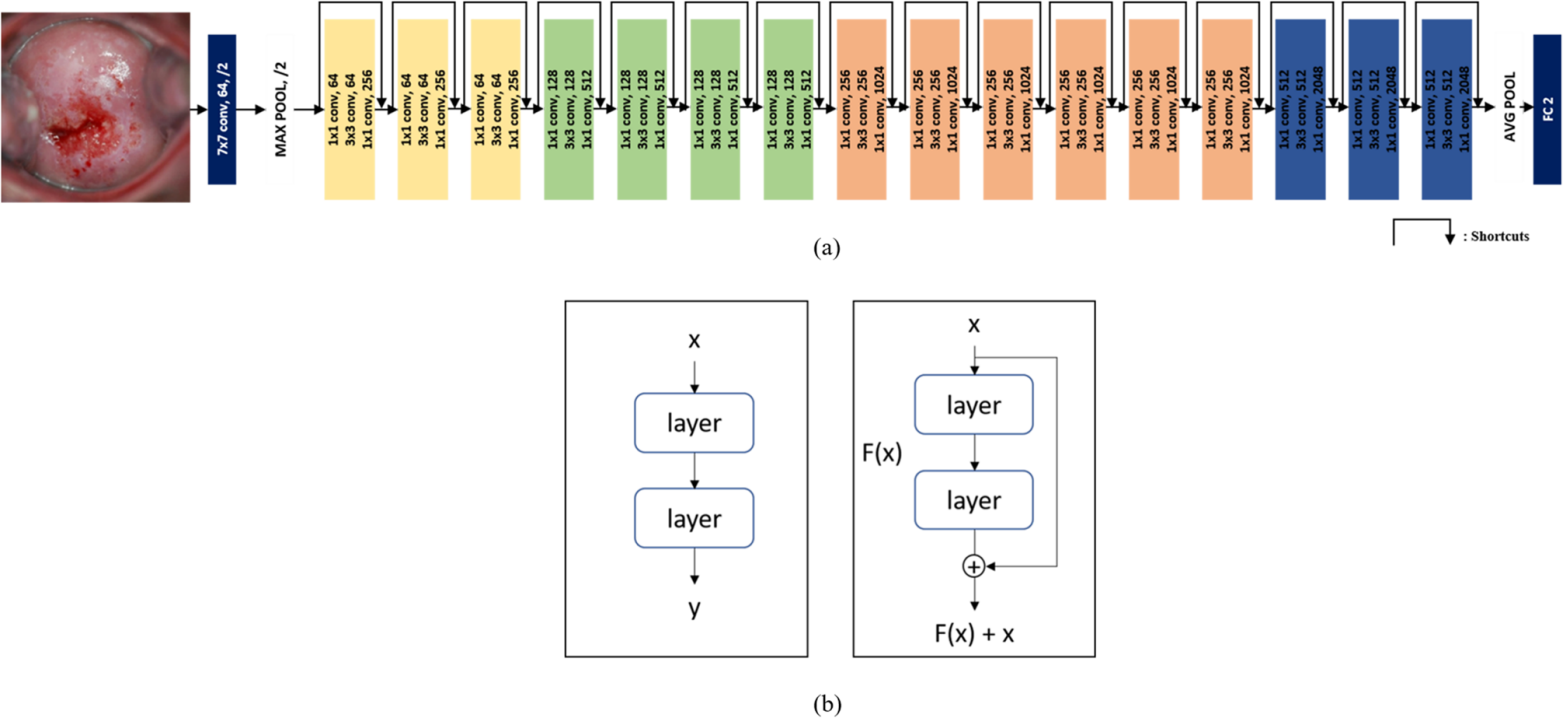
(**a**) ResNet50 Architecture of DL, reducing the dimension by adding a 1 × 1 conv to each layer, the amount of computation is reduced and the training speed is accelerated. (**b**) learning method of existing CNN (left) and ResNet (right). By adding shortcuts that add input values to output values every two layers, errors are reduced faster than with existing CNNs.

For DL, we used the ResNet-50 algorithm, a type of deep convolution neural network (DCNN) (Fig. 4a). As shown in Fig. 4b, the traditional CNN method was used to find the optimal value of input x through the learning layer, while ResNet-50 was used to find the optimal F(x) + x by adding input x after the learning layer. This approach has advantages in reducing both network complexity and the vanishing gradient problem, resulting in faster training.^29^.

We then applied a transfer-learning technique using a network pre-trained on ImageNet^30^. At the beginning of training, the weights of the pre-trained layers are frozen, and at the point where the loss no longer falls during training, the newly added layer is judged to be well trained, and the weights of all layers are made trainable and training is resumed. Parameters for training were set to batch size of 40 and 300 epochs, which was suitable for the computing power of the hardware. The learning rate was set to be 0.0001 to prevent significant changes in transition learning weights. To improve learning speed, the images were resized to 256 × 256.

### Evaluation process

Cross validation is an evaluation method that prevents overfitting and improves accuracy when evaluating model performance. We validated the classification performance of two algorithms with a fivefold cross validation, a method in which all datasets were tested once each with a total of five verifications. To ensure comparable results, the same five training sets and test sets were used in each method.

Usually binary classifiers are evaluated based on basic decision scores. The scores include True positive (TP), the number of positive samples correctly classified as positive, True negative (TN), the number of negative samples correctly classified as negative, False positive (FP), the number of negative samples incorrectly classified as positive, and False negative (FN), the number of positive samples incorrectly classified as negative. Using the above scores, we evaluated each model with metrics as follows. Precision, also known as PPV, is the ratio of what is true to what is classified as true. Recall, which is also known as sensitivity, is the ratio of what the model predicts to be true among what is true. The F1-score is the harmonic mean of precision and recall. Accuracy is the proportion of the total predicted trues that are true, and the proportion of the total predicted false classifications that are false.

1 $$\mathrm{Precision}=\frac{TP}{TP+FP}$$

2 $$\mathrm{Recall}=\frac{TP}{TP+FN}$$

3 $$\mathrm{F}1\mathrm{ score}=\frac{2 * precision * recall}{precision+recall}$$

4 $$\mathrm{Accuracy}=\frac{TP + TN}{TP + FN+TN+FP}$$

## Results

### Visualization

**Figure 5:**
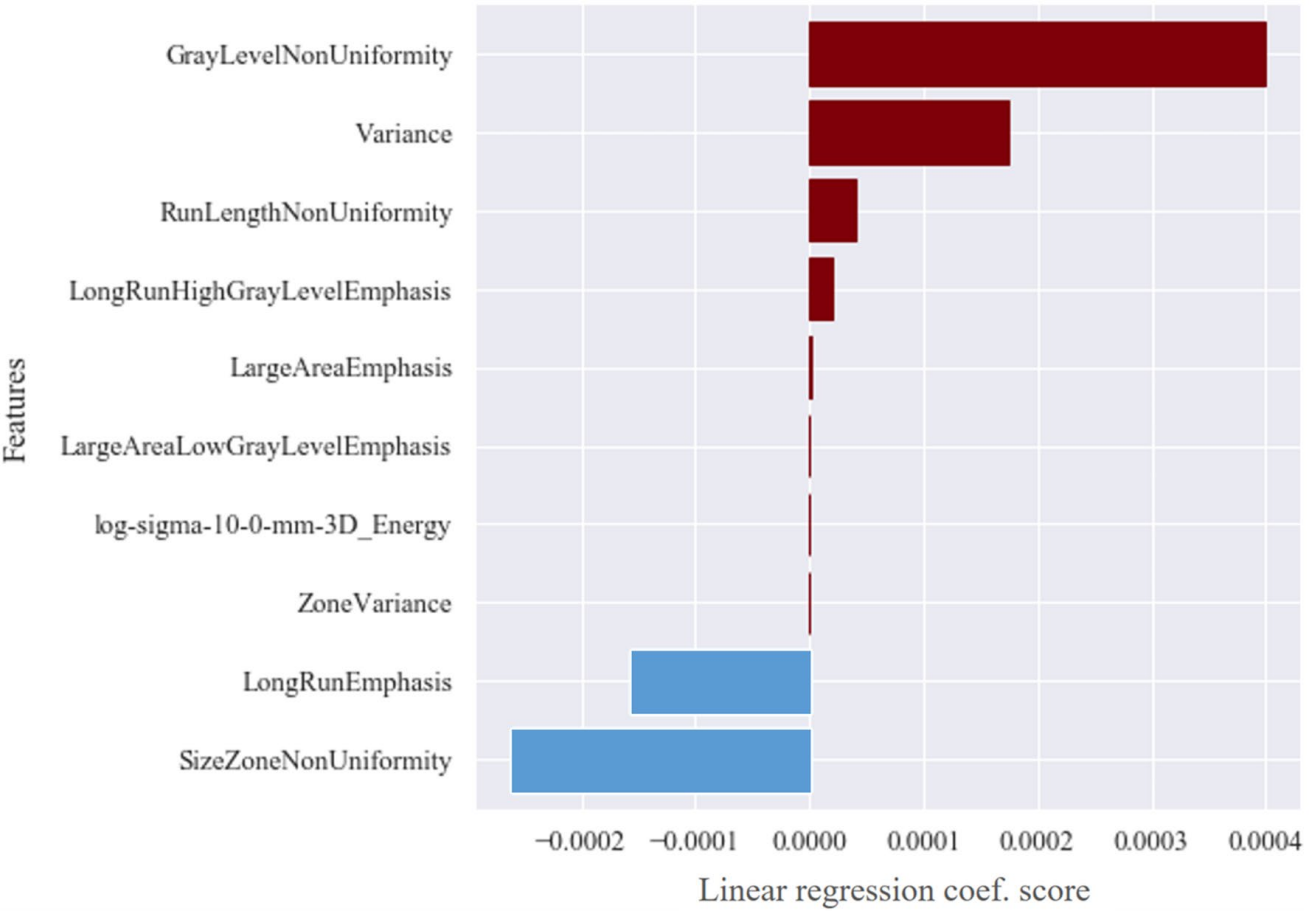
Selected 10 features by Lasso regression efficient score. 8 features showed positive coefficient scores (red) while 2 features showed negative coefficient scores (blue).

The bar graph in Fig. 5 shows the 10 selected features and the importance of each feature to the ML models. Features with values greater than zero have positive linear relationships while features smaller than zero have negative linear relationships.

**Figure 6:**
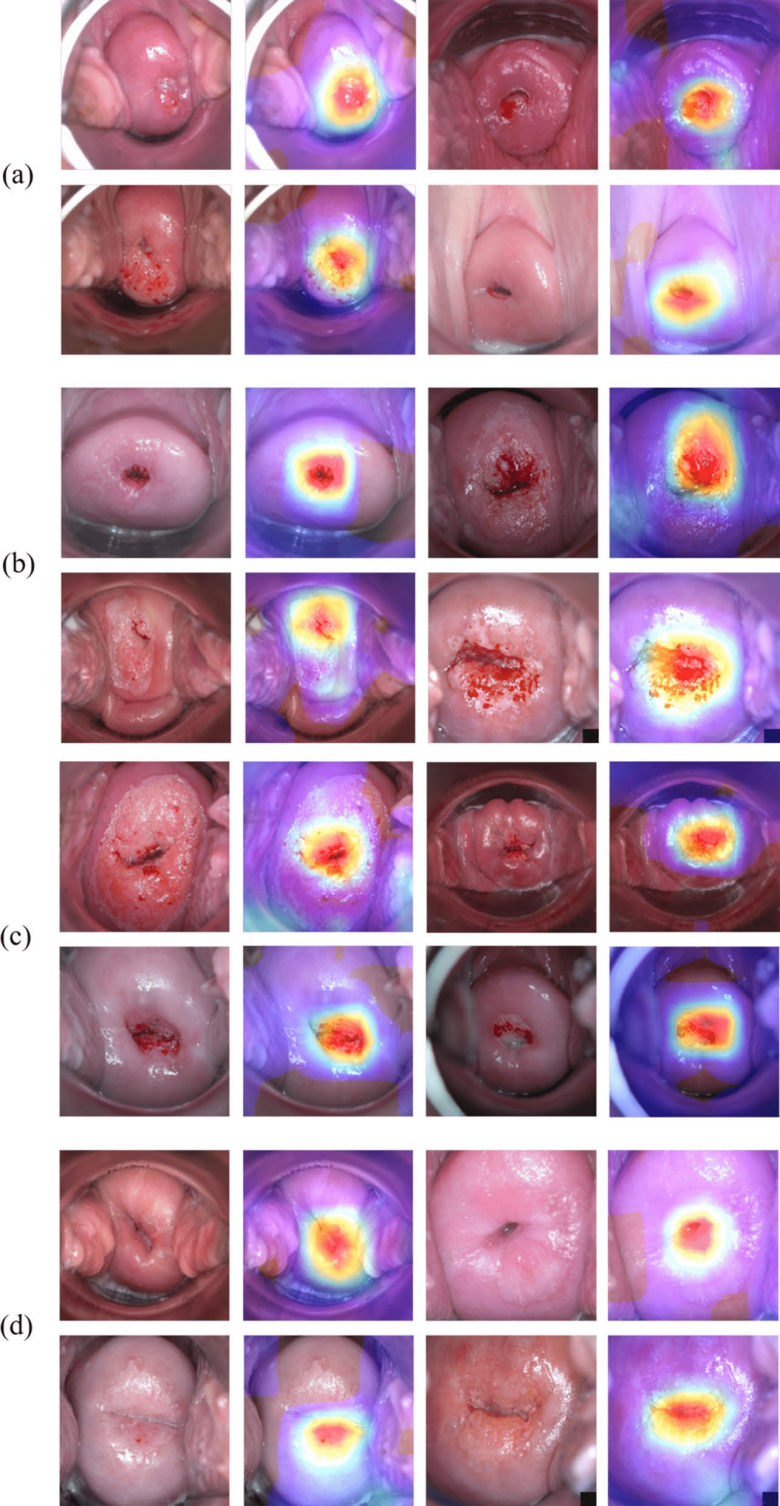
Examples of CAM images of test sets. (**a**) True Negative (ground truth: negative, predict: negative) (**b**) True Positive (ground truth: positive, predict: positive) (**c**) False Positive (ground truth: negative, predict: positive) (**d**) False Negative (ground truth: positive, predict: negative).

To determine which area the DL recognized as negative or positive, results from the test set were visualized using a Class Activation Map (CAM) to show which areas were given more weight (Fig. 6).

### Evaluation

**Figure 7:**
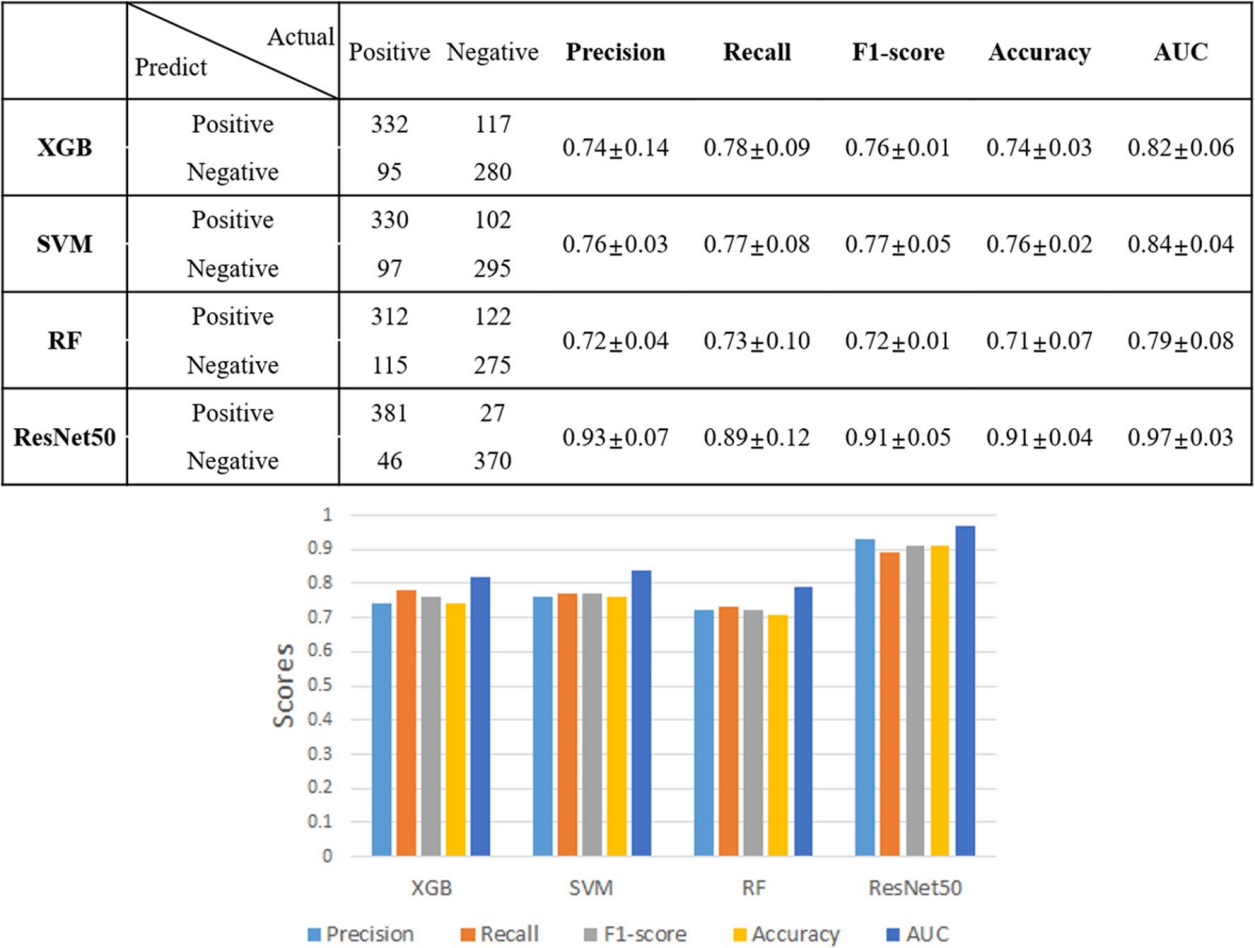
Internal cross validation of cervical cancer predictions. ResNet-50 showed the highest performance in all metrics. SVM showed the highest performance when compared with the AUCs of the ML models, excluding ResNet-50.

To evaluate the performance of the ML and DL models, results were validated by fivefold cross validation using precision, recall, f1-score, and accuracy indicators as shown in Fig. 7.

**Figure 8:**
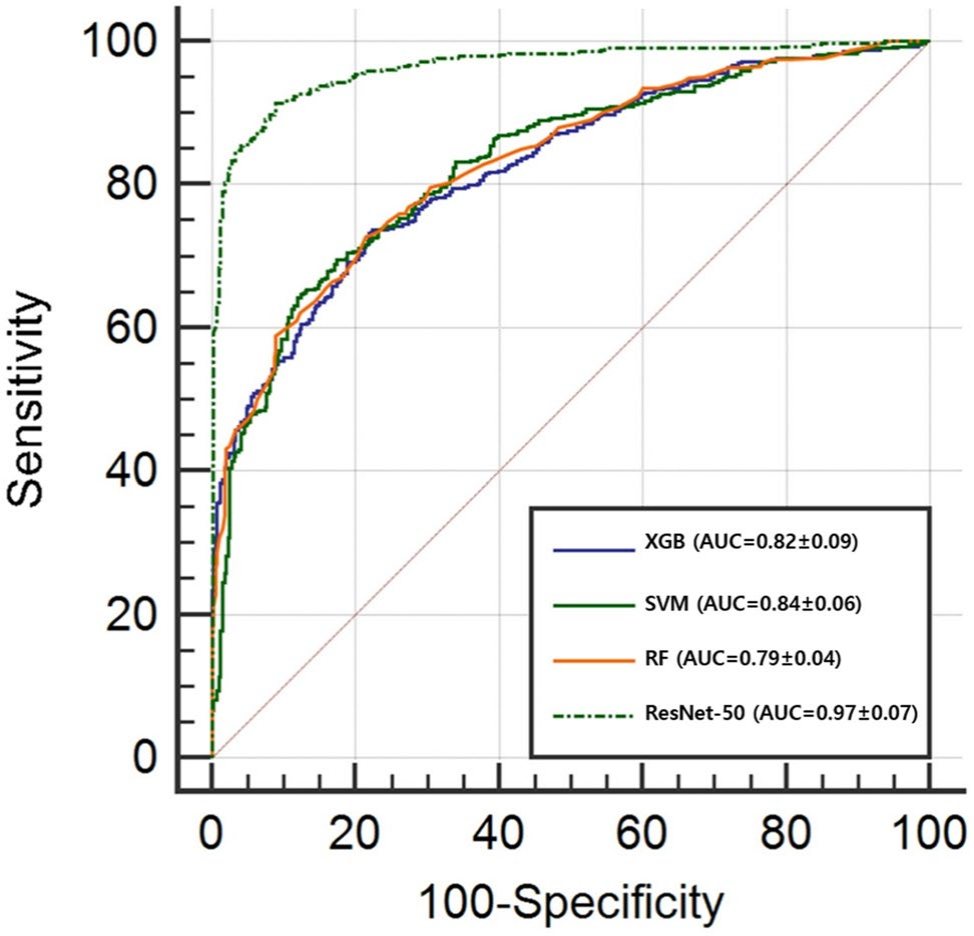
Mean ROC comparison graph of fivefold cross validation of each method to predict cervical cancer.

ResNet-50 had the highest AUC at 0.97 (95% confidence interval [CI]: 94.9–97.6%). SVM followed with an AUC of 0.84 (95% CI: 80.1–85.4%). XGB was very similar to SVM with an AUC of 0.82 (95% CI: 79.7–85.1%). RF had the lowest AUC at 0.79%. ResNet-50’s AUC was 0.15 points higher than the average (0.82) of the three ML algorithms (*p* < 0.05) (Fig. 8).

## Discussion

### Principal findings

In this study, we compared the performance of ML and DL models by automatically classifying cervical images as negative or positive for signs of cervical cancer. The ResNet-50 architecture’s performance was 15% higher than the average performance of the XGB, RF, and SVM models.

### Results

Herein, we investigated the performance of ML and DL models to determine which algorithm would be more suitable to assist clinicians with the accurate diagnosis of cervical cancer. Using 1984 negative images and 2135 positive images, a total of 4119 cervicography images were used to select 10 out of 300 features from pre-processed images in a linear regression. Three algorithms (XGB, SVM, and RF) were used to create the ML classification models. The DL classification model with ResNet-50 architecture was also generated using the same pre-processed images. With both ML and DL techniques, our assessment found more reliable results when all datasets were tested using fivefold cross validation. The AUC values for XGB, SVM, and RF were 0.82, 0.84, and 0.79, respectively. Resnet-50 showed an AUC value of 0.97. ML algorithms did not exceed 0.80 for accuracy, while ResNet-50 showed an accuracy of 0.9065 with a relatively better performance.

### Clinical implications

Generally, when diagnosing cervical cancer in clinical practice, lesions are diagnosed by compiling several data points including the thickness of the aceto-white area, presence of transformation zone, and tumor identification. Given the complexity of the diagnostic process, the end-to-end method of DL, which divides the problem into multiple parts and obtains answers for each and outputs results considering comprehensively each answers, likely contributed to the DL model’s improved performance in the cervical cancer classification task in the learning system. Compared to DL, ML splits the problem into multiple parts, obtains the answers for each, and just adds the results together. We speculate that the step-by-step methods of ML may have had difficulty understanding and learning these complex diagnostic processes.

### Research implications

In terms of algorithms, DL identifies and learns meaningful features from the totality of features by itself, while ML requires that unnecessary features be removed by human experts before training. This difference could be responsible for the decreased performance of the ML models. Since DL learns low-level features in the initial layer and high-level features as the layers deepen, the weight of high-level features, which are not learned by ML, is likely responsible for the difference in performance between the two types of systems. Thus, DL likely performed better due to its integration of high-level features.

In this study, cervical images were cropped to create a uniform dataset as was needed for the ML models and to provide the basis for an accurate comparison between the DL and ML architectures. In future research, the addition of a DL-based cervical detection model to this classification task could further improve the accuracy of model comparisons by facilitating the selection of only the appropriate areas to be analyzed.

### Strengths and limitations

This study is the first to compare the performance of DL and ML in the field of automatic cervical cancer classification. Compared to other studies that have produced results using only one method, either DL or ML, this work enables cervical clinicians to objectively evaluate which automation algorithms are likely to perform better as computer-aided diagnostic tools.

In pre-processing, the same width was cropped from both ends of the image to remove vaginal wall areas, assuming that the cervix was exactly in the middle. However, not all images had the cervix in the center or the shape of the cervix has possibility to be distorted and out of the desired area. The cropped images we used may still have contained vaginal walls, which are unnecessary, or the cervix that was intended to be analyzed could have been cropped out. This may have disproportionally decreased the accuracy of one model or the other, weakening the comparison.

In addition, Data augmentation is known to help increase generalization of networks and help reduce overfitting during training. It is thought that it will be possible to compare models with higher performance by applying this data augmentation method in future studies.

Moreover, when selecting ML features, the lasso technique was used and 10 features were selected. However, adopting a different feature selection method or selecting features more or less than 10 could result in a completely different outcome. The fact that human intervention is involved in the ML process itself is a major disadvantage and could mean that it is not possible to truly compare ML to DL models accurately.

## Conclusion

Herein, the performance of ML and DL techniques was objectively evaluated and compared via the classification of cervicography images pre-processed by the same methods.

The results of this research can serve as a criterion for the objective evaluation of which techniques will likely provide the most robust computer-assisted diagnostic tools in the future.

Furthermore, when diagnosing cervical cancer, it may be clinically relevant to consider the diagnostic factors identified by multiple model architectures.

In future studies, a more accurate comparison of cervical cancer classification performance could be conducted by adding a detection model that accurately detects and analyzes only the cervix. Finding and adopting better techniques for feature selection could also minimize human intervention in ML, strengthening the comparison between different model architectures. We expect these future studies to allow for a more objective comparison of different model architectures that will ultimately assist clinicians in choosing appropriate computer-assisted diagnostic tools.

## References

[1] Steven EW (2003). Cervical cancer. Lancet.

[2] Rebecca LS (2019). Cancer Statistics 2019. Cancer J. Clin..

[3] Canfell K (2020). Mortality impact of achieving WHO cervical cancer elimination targets: A comparative modelling analysis in 78 low-income and lower-middle-income countries. Lancet.

[4] Adolf S (1981). Cervicography: A new method for cervical cancer detection. Am. J. Obstet. Gynecol..

[5] Janicek MF (2008). Cervical cancer: Prevention, diagnosis, and therapeutics. Cancer J. Clin..

[6] Mandelblatt JS (2002). Costs and benefits of different strategies to screen for cervical cancer in less-developed countries. J. Natl. Cancer Inst..

[7] Wright TCJR (2007). Cervical cancer screening in the 21st century: Is it time to retire the PAP smear?. Clin. Obstet. Gynecol..

[8] Ottaviano M (1982). Examination of the cervix with the naked eye using acetic acid test. Am. J. Obstet. Gynecol..

[9] Small W (2017). Cervical cancer: A global health crisis. Cancer J. Clin..

[10] Schiffman M (2005). The promise of global cervical-cancer prevention. N. Engl. J. Med..

[11] Sezgin MI (1989). Observer variation in histopathological diagnosis and grading of cervical intraepithelial neoplasia. Br. Med. J..

[12] Sigler DC (1985). Inter- and intra-examiner reliability of the upper cervical X-ray marking system. J. Manip. Physiol. Ther..

[13] Richard L (2007). Comparison of computer-assisted and manual screening of cervical cytology. Gynecol. Oncol..

[14] Afzal HS (2020). A deep learning approach for prediction of Parkinson’s disease progression. Biomed. Eng. Lett..

[15] Alexzandru K (2017). Comparison of deep learning with multiple machine learning methods and metrics using diverse drug discovery data sets. Mol. Pharm..

[16] Kim KG (2016). Deep learning. Healthc. Inf. Res..

[17] Xiaoyu, D., et al. Analysis of risk factors for cervical cancer based on machine learning methods. In *Proceedings of the IEEE International Conference on Cloud Computing and Intelligence Systems (CCIS)*, 631–635 (IEEE, 2018).

[18] Héctor-Gabriel A (2009). Aceto-white temporal pattern classification using k-NN to identify precancerous cervical lesion in colposcopic images. Comput. Biol. Med..

[19] Muhammad, T., et al. Classification of colposcopy data using GLCM-SVM on cervical cancer. In *Proceedings of 2020 International Conference on Artificial Intelligence Information and Communication* 373–378 (ICAIIC, 2020).

[20] Kim HY (1999). A study on nucleus segmentation of uterine cervical pap-smears using multi stage segmentation technique. J. Korean Soc. Med. Inf..

[21] Limming H (2019). An observational study of deep learning and automated evaluation of cervical images for cancer screening. JNCI.

[22] Masakazu S (2018). Application of deep learning to the classification of images from colposcopy. Oncol. Lett..

[23] Kangkana, B., et al. Pap smear image classification using convolutional neural network. In *ICVGIP ‘16* Vol. 55, 1–8 (2016).

[24] Valeria F (2017). Feature selection using lasso. VU Amsterdam Res. Pap. Bus. Anal..

[25] Robert M (1973). Textural Features for Image Classification. IEEE Trans. Syst. Man. Cybern..

[26] Tianqi, C., et al. Xgboost: A scalable tree boosting system. In *Proceedings of the 22nd ACM SIGKDD International Conference on Knowledge Discovery and Data Mining* 785–794 (KDD, 2016).

[27] Andy L (2002). Classification and regression by randomForest. R News.

[28] William SN (2006). What is a support vector machine?. Nat. Biotechnol..

[29] Sasha, T., et al. Resnet in resnet: Generalizing residual architectures. arXiv Prepr. arXiv1603.08029 (2016).

[30] Raja, R., et al. Self-taught learning: transfer learning from unlabeled data. In *Proceedings of the 24th International Conference on Machine Learning* 759–766 (ICML, 2017).

